# Evaluation of mRNA Biomarkers to Identify Risk of Hospital Acquired Infections in Children Admitted to Paediatric Intensive Care Unit

**DOI:** 10.1371/journal.pone.0152388

**Published:** 2016-03-25

**Authors:** Estelle Peronnet, Kha Nguyen, Elisabeth Cerrato, Rathi Guhadasan, Fabienne Venet, Julien Textoris, Alexandre Pachot, Guillaume Monneret, Enitan Delphine Carrol

**Affiliations:** 1 Joint Research Unit Hospices Civils de Lyon / bioMérieux, Hôpital Edouard Herriot—Pathophysiology of injury induced immunosuppression (PI3) Lab, Lyon 1 University / Hospices Civils de Lyon / bioMérieux, Lyon, France; 2 Department of Women’s and Children’s Health, Institute of Translational Medicine, University of Liverpool, Liverpool, United Kingdom; 3 Department of Clinical Infection, Microbiology and Immunology, Institute of Infection and Global Health, University of Liverpool, Liverpool, United Kingdom; 4 Immunology Laboratory, Hôpital Edouard Herriot, Hospices Civils de Lyon, Lyon, France; 5 Anesthesiology and Intensive Care department, Hôpital Edouard Herriot, Hospices Civils de Lyon, Lyon, France; Centro de Pesquisa Rene Rachou/Fundação Oswaldo Cruz (Fiocruz-Minas), BRAZIL

## Abstract

**Objectives:**

Hospital-acquired infections (HAI) are associated with significant mortality and morbidity and prolongation of hospital stay, adding strain on limited hospital resources. Despite stringent infection control practices some children remain at high risk of developing HAI. The development of biomarkers which could identify these patients would be useful. In this study our objective was to evaluate mRNA candidate biomarkers for HAI prediction in a pediatric intensive care unit.

**Design:**

Serial blood samples were collected from patients admitted to pediatric intensive care unit between March and June 2012. Candidate gene expression (*IL1B*, *TNF*, *IL10*, *CD3D*, *BCL2*, *BID*) was quantified using RT-qPCR. Comparisons of relative gene expression between those that did not develop HAI versus those that did were performed using Mann Whitney U-test.

**Patients:**

Exclusion criteria were: age <28 days or ≥16 years, expected length of stay < 24 hours, expected survival < 28 days, end-stage renal disease and end-stage liver disease. Finally, 45 children were included in this study.

**Main Results:**

The overall HAI rate was 30% of which 62% were respiratory infections. Children who developed HAI had a three-fold increase in hospital stay compared to those who did not (27 days versus 9 days, *p*<0.001). An increased expression of cytokine genes (*IL1B* and *IL10*) was observed in patients who developed HAI, as well as a pro-apoptosis pattern (higher expression of *BID* and lower expression of *BCL2*). *CD3D*, a key TCR co-factor was also significantly down-modulated in patients who developed HAI.

**Conclusions:**

To our knowledge, this is the first study of mRNA biomarkers of HAI in the paediatric population. Increased mRNA expressions of anti-inflammatory cytokine and modulation of apoptotic genes suggest the development of immunosuppression in critically ill children. Immune monitoring using a panel of genes may offer a novel stratification tool to identify HAI risk.

## Introduction

Prolonged inflammatory stimuli associated with critical illness contribute to innate and adaptive immune dysfunctions, leading to susceptibility to hospital-acquired infection (HAI) [1,2]. The phenomenon of ICU-acquired immune dysfunction is called compensatory anti-inflammatory response syndrome [3]. The proposed mechanisms following initial insult include T-cell anergy, endotoxin tolerance, apoptosis of immune cells [4–6], anti-inflammatory mediator production, and epigenetic regulation [7].

HAI is a significant burden on hospital resources resulting in prolonged intensive care unit (ICU) stay and ventilator-dependent days. It directly causes 5,000 deaths per year in the United Kingdom and contributes to 15,000 death cases [8,9]. Children with indwelling devices, such as in ICU, are at increased risk of developing HAI. Published work on adult and paediatric patients admitted to ICU has identified quantifiable immune dysfunctions in severe sepsis and trauma, and consequently its association to the development of HAI [1,10–13].

In children, like in critically ill adults, immunosuppression is potentially reversible using immunostimulatory drugs. Several candidates targeting both the innate (GM-CSF, IFNγ) and the adaptive (IL-7, monoclonal antibodies against PD1/PDL1) immune responses are currently good candidates to restore the immune response in these patients. Early recognition of immunosuppression is important to select patients at high risk of HAI that could benefit from novel therapeutic strategies and could be achieved using mRNA biomarkers. Recently, fully automated, multiplexed and standardized quantitative PCR platforms were developed. This offers the possibility to validate and transfer molecular biomarkers in routine clinics. This study aimed to determine the utility of mRNA biomarkers for risk stratification in critically ill children at risk of HAI.

## Materials and Methods

### Patients recruitment

Between March and June 2012, children from birth to 16 years admitted to paediatric intensive care unit (PICU) in Alder Hey Children’s Hospital were screened in this study. Exclusion criteria were <28 days or ≥16 years of age, children admitted moribund and not expected to survive more than 24 hours, children who were non-intubated elective admissions with a predicted duration of stay of less than 24 hours, children not expected to survive at least 28 days because of pre-existing condition, presence of existing directive to withhold life-sustaining treatment, end-stage renal disease requiring chronic dialysis therapy, end-stage liver disease: cirrhosis with evidence of portal hypertension and congenital immunodeficiency. Patients aged less than 28 days were excluded from the study because modulation of the immune response is different in infants under 28 days compared to older infants and children [14].

HAI was defined according to CDC criteria as a localized or systemic condition resulting from an adverse reaction to the presence of an infectious agent(s) or its toxin(s) that was not present on admission to the acute care facility. An infection was considered an HAI if all elements of a CDC and Prevention/National Healthcare Safety Network site-specific infection criterion were not present during the period of admission but were all present on or after the 3^rd^ calendar day of admission to the facility (the day of hospital admission is calendar day 1) [15].

The protocol was approved by the National Research Ethics Service reference 10/H1014/52 and parents gave their written informed consent to the study.

### Sample collection

Blood samples for gene expression analysis were collected using a modified protocol adapted for low blood volumes [16]. Peripheral whole blood (0.5 mL) from venipuncture was dispensed into 2 mL cryogenic tubes pre-aliquoted with 1.38 mL PAXgene^TM^ reagent (PreAnalytix, Hilden, Germany), keeping the blood:reagent ratio the same as in the PAXgene^TM^ Blood RNA tubes. Two samples were collected for each patient: on day 1 and between day 2 and day 4 after PICU admission, before HAI onset. Samples were stored at -80°C within 2 hours of collection.

### RNA extraction, quality control and reverse transcription

Total RNA was extracted from whole blood using PAXgene^TM^ Blood RNA Kit (PreAnalytix, Hilden, Germany), employing an amended version of the manufacturer’s guidelines: after the first centrifugation, the pellet was washed with 0.8 mL of DNAse free water, in order to keep the same ratio as in the initial method. Before RNA elution, the residual genomic DNA was digested using the Rnase-Free Dnase set (Qiagen, Hilden, Germany).

RNA integrity was assessed with the RNA 6000 Nano Kit on a Bioanalyzer (Agilent Technologies, Santa Clara, California). Total RNA was reverse transcribed in complementary cDNA using SuperScript® VILO™ cDNA Synthesis Kit (Life Technologies, Chicago, IL).

### Real time quantitative polymerase chain reaction

A panel of six genes involved in the host response to injury was chosen. They could be classified in three groups: 1) *IL1B* and *TNF* are known to be pro-inflammatory cytokines; 2) *CD3D* and *IL10* are known to be involved in immunosuppression; 3) *BCL2* and *BID* are involved in lymphocyte apoptosis. The expression of the panel of genes (genes of interest and reference genes) was quantified using q-real time polymerase chain reaction (PCR). PCR was performed in a LightCycler instrument using the standard Taqman Fast Advanced Master Mix PCR kit according to the manufacturer’s instructions (Roche Molecular Biochemicals, Basel, Switzerland). Thermocycling was performed in a final volume of 20 μL containing 0.5 μM of primers and 0.1 μM of probe. Primers and probes designs for candidate and reference genes are listed in S1 Table, except for *BID* and *BCL2* primers and probes that were provided by Applied Biosystems (Life Technologies, Chicago, IL). PCR was performed with an initial denaturation step of 10 min at 95°C, followed by 45 cycles of a touchdown PCR protocol (10 sec at 95°C, 29 sec annealing at 68–58°C, and 1 sec extension at 72°C). The Second Derivative Maximum Method was used with the LightCycler software to automatically determine the crossing point for individual samples. Standard curves were generated by using four replicates of cDNA standards and were used to perform efficiency corrected quantification.

Gene expression normalization was performed based on the combination of two selected reference genes (*HPRT1*: hypoxanthine phosphoribosyltransferase 1 and *PPIB*: peptidylprolyl isomerase B) and results were expressed as Calibrated Normalized Relative Quantity [17]. Both reference genes were selected among a list of six candidates. The selection was performed using the tools available via RefFinder (available from: http://www.leonxie.com/referencegene.php).

### Statistics

Only samples collected before HAI onset were considered in the analysis. Comparisons between groups (No HAI vs HAI) were made using the non-parametric Mann Whitney U-test for continuous variables and the Fisher’s exact test for categorical data. Values of *p*<0.05 were considered statistically significant. Receiver operating characteristic curves and areas under curve (AUC) were calculated for each candidate marker, as well as *p-values* that test the null hypothesis that the area under the curve equals 0.50. Correlations were performed using Spearman test. Correlations with r>0.8 were considered significant. Statistical analyses were performed with GraphPad Prism® software (version 5.02, GraphPad Software, La Jolla, CA).

## Results

### Study population and hospital-acquired infections description

**Fig 1:**
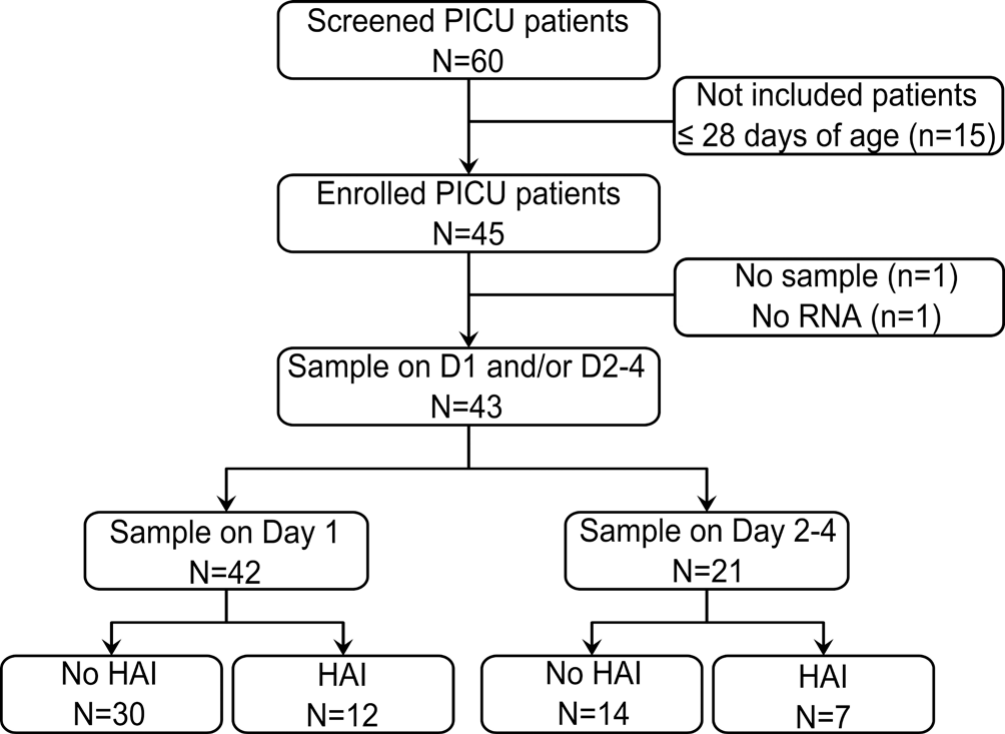
Study flowchart. A total of 60 patients were screened. Fifteen patients less than 28 days of age were excluded. One patient had no sample on day 1 nor day 2–4 and one patient had samples with poor-quality RNA. These two patients were excluded for technical reasons. PICU: paediatric intensive care unit; HAI: hospital-acquired infections

Forty five patients from 1 month to 14 years old admitted to PICU were enrolled in this study (see flowchart on Fig 1). Two samples were discarded for technical reasons. Therefore we analyzed a cohort of 43 patients, whose clinical characteristics are described in Table 1. We observed a relatively low severity in this cohort, with a median PELOD score of 11 and only 2 deceased patients.

**Table 1:** Characteristics of the 43 paediatric patients according to hospital-acquired infection occurrence.

Variable	HAI (N = 13)	No HAI (N = 30)	Total (N = 43)	*p* value
**Demographics**				
Gender–Male, n (%)	6 (46)	13 (43)	19 (44)	0.87
Age (Months), median [IQR]	16 [9–64]	15 [6–66]	15 [7–66]	0.63
**Admission data**				
*Reason for admission*				
Cardiac surgery, n (%)	8 (62)	16 (53)	24 (56)	0.49
Other surgery, n (%)	0 (0)	3 (10)	3 (7)	
Sepsis, n (%)	2 (15)	8 (27)	10 (23)	
Other, n (%)	3 (23)	3 (10)	6 (14)	
*Comorbidities*				
Congenital heart disease, n (%)	2 (15)	11 (37)	13 (30)	0.28
Non cardiac congenital disease, n (%)	1 (8)	3 (10)	4 (9)	1.00
Chromosomal abnormality, n (%)	7 (54)	4 (13)	11 (26)	**0.01**
PELOD, median [IQR]	12 [11–12]	11 [7–12]^a^	11 [11–12]^b^	0.27
Cardiopulmonary bypass (CPB), n (%)	8 (62)	16 (53)	24 (56)	0.74
Dexamethasone (Pre, post, during CPB), n(%)	5 (63)^c^	10 (63)^d^	15 (63)^e^	1.00
PIM2 score, median [IQR]	0.042 [0.016–0.078]^c^	0.050 [0.028–0.074]^d^	0.047 [0.022–0.078]^e^	0.35
**Treatments**				
ATB prior to admission, n(%)	7 (54)	14 (52)^a^	21 (53)^b^	0.83
ATB during admission, n(%)	13 (100)	30 (100)	43 (100)	1.00
Blood transfusion, n(%)	7 (54)	14 (48)^f^	21 (50)^g^	1.00
**Biological data day 1**				
WCC (10^9^/L), median [IQR]	16 [11–20]	11 [8.5–12]	11 [8.5–16]	**0.04**
Lymphocytes (10^9^/L), median [IQR]	1.1 [0.9–1.6]	1.5 [1.0–2.6]	1.4 [0.9–2.6]	0.23
Lactate (mmol/L), median [IQR]	1.8 [1.0–2.1]^h^	1.0 [0.7–1.4]^i^	1.1 [0.8–2.0]^j^	0.20
C reactive protein (mg/L)	25 [4–65]^k^	14 [4–31]	17 [4–63]^g^	0.64
**Biological data day 2–4**				
WCC (10^9^/L), median [IQR]	11 [9.8–21]^l^	11 [7.7–14]^m^	11 [8.9–15]^n^	0.11
Lymphocytes (10^9^/L), median [IQR]	2.9 [2.2–3.3]^l^	2.3 [1.6–4.0]^m^	2.4 [1.7–3.6]^n^	0.79
C reactive protein (mg/L), median [IQR]	25 [18–59]^l^	23 [9.2–76]^m^	23 [12–64]^n^	0.74
**Risk factors**				
*Invasive devices at admission*				
Intubation, n (%)	12 (92)	29 (97)	41 (95)	0.52
Central venous line, n (%)	11 (85)	21 (70)	32 (74)	0.46
Urinary catheter, n (%)	11 (85)	26 (87)	37 (86)	1.00
**Outcomes**				
Mortality, n (%)	0 (0)	2 (7)	2 (5)	1.00
ICU length of stay (Days), median [IQR]	4 [3–14]	4 [2–6]	4 [2–6]	0.11
Hosp. length of stay (Days), median [IQR]	27 [12–31]	9 [6–18]	12 [6–21]	**<0.001**
Ventilation duration (Days), median [IQR]	5 [2–11]	3 [1–6]	3 [2–7]	0.08

^a^ n = 27

^b^ n = 40

^c^ n = 8

^d^ n = 16

^e^ n = 24

^f^ n = 29

^g^ n = 42

^h^ n = 11

^i^ n = 28

^j^ n = 39

^k^ n = 12

^l^ n = 7

^m^ n = 14

^n^ n = 21.

HAI and no HAI groups were compared using Mann-Whitney test for continuous variables and Fisher’s exact test for categorical variables. *p* values <0.05 are bold.

HAI: Hospital-Acquired Infection. Hosp: hospital. PELOD: Pediatric Logistic Organ Dysfunction. CPB: Cardiopulmonary bypass. PIM2: Pediatric Index for Mortality. ATB: antibiotics. WCC: White cells count. ICU: Intensive Care Unit.

In this study, overall HAI rate was 30% (n = 13). Ten patients (77%) had single HAI event whereas three patients (22%) had two separate HAI events. As shown in Table 2, the median time to onset of first infection episode was 6 days. Infection occurred in PICU for 5 patients and during hospital stay after PICU discharge for 8 patients. Most HAI were pulmonary infections (62%).

**Table 2:** Characteristics of hospital-acquired infections.

**Number of patients with HAI**	13
**Number of HAI episode/ patient**	
1, n (%)	10 (77)
2, n (%)	3 (23)
**Delay of 1**^**st**^ **HAI occurrence (Days),** median [IQR]	6 [4–10]
**Site of 1**^**st**^ **HAI**	
Respiratory / pulmonary, n (%)	8 (62)
Device-associated infections, n (%)	2 (15)
Urinary tract infections, n (%)	1 (8)
Undetermined, n (%)	2 (15)
**Type of pathogen causing 1**^**st**^ **HAI**	
Bacteria, n (%)	4 (31)
Virus, n (%)	3 (23)
Unknown, n (%)	6 (46)

HAI: Hospital-Acquired Infection

The main reason for admission was cardiac surgery (56%) and there were only 10 patients who initially presented with sepsis on PICU admission. We observed a similar rate of secondary infections in the medical (5/16, 31%) and the surgical (8/27, 30%) patients. There was no statistically significant difference between HAI and no HAI patients regarding demographic and admission data. White blood cell count at day 1 was higher in patients who developed HAI (16.10^9^ cells/L [11–20] *vs* 11.10^9^ cells/L [8.5–12]; *p* = 0.04). We observed no differences between groups regarding invasive devices, known as HAI risk factors.

As expected, children who developed HAI had a significantly increased duration of hospital stay than those who did not (27 [12–31] *vs*. 9 [6–18] days, respectively; *p*<0.001).

At day 2–4, 21 patients were still in study. This high attrition rate is due to (1) the occurrence of HAI before day 2–4 for two patients who were not analyzed for the second time point and (2) patient discharge or central line removal for 19 patients between day 2 and day 4. As shown in S2 Table, clinical characteristics of the 21 patients with sample at day 2–4 were similar to those for all patients with sample on day 1, with higher hospital length of stay for children who contracted HAI.

### Evaluation of mRNA candidate gene levels for the identification of patients at risk of HAI

**Fig 2:**
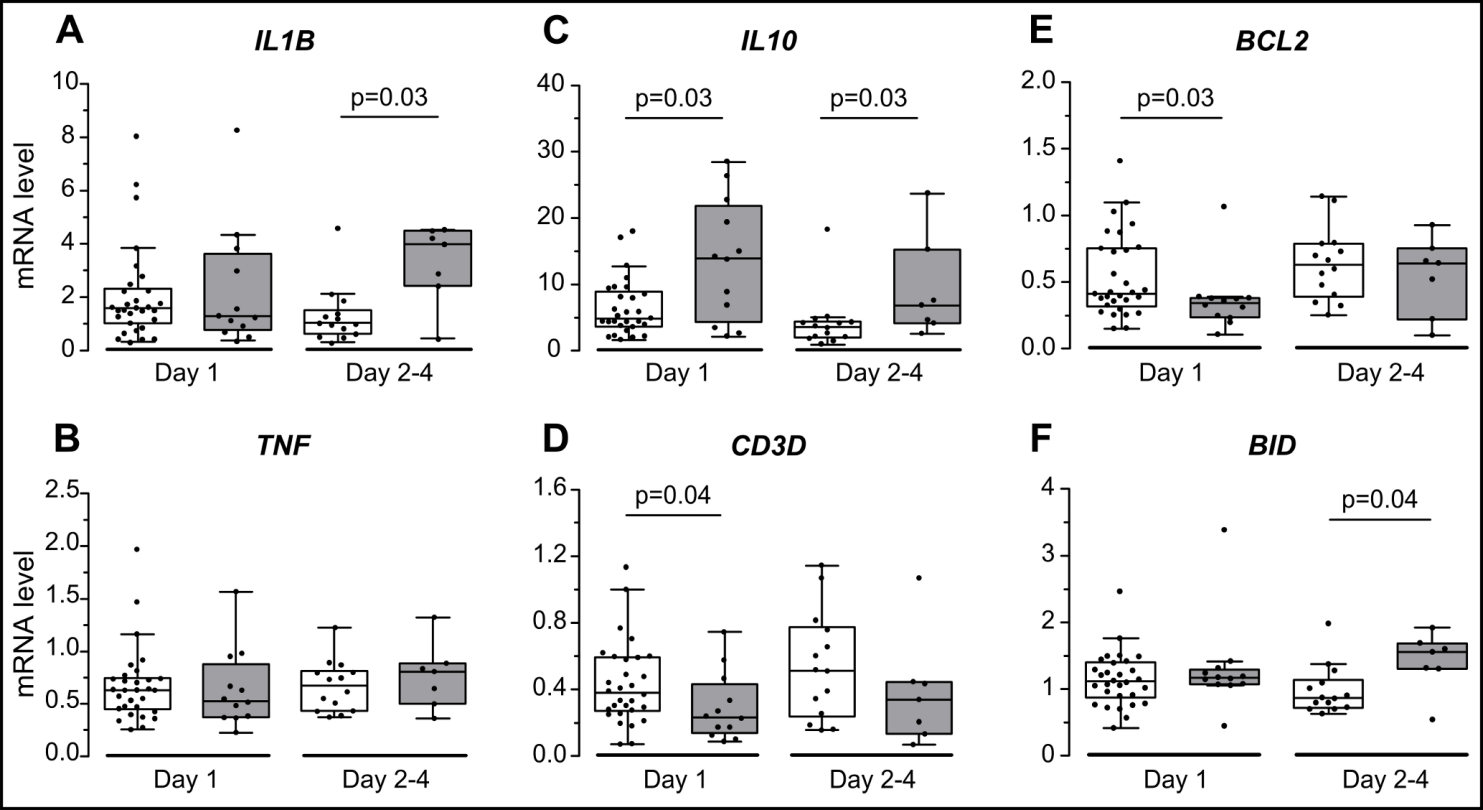
Comparison of gene expression levels between HAI and no HAI patients. No HAI patients (Clear): n = 30 for day 1 and n = 14 for day 2–4 (except IL10: n = 29 for day 1); HAI patients (Grey): n = 12 for day 1 and n = 7 for day 2–4. Gene expression levels of (A) *IL1B*, (B) *TNF*, (C) *IL10*, (D) *CD3D*, (E) *BCL2* and (F) *BID* are expressed as Calibrated Normalized Relative Quantity using *PPIB* and *HPRT1* as reference genes. No HAI and HAI groups were compared using Mann-Whitney test and *p* <0.05 are indicated on plots. HAI: hospital-acquired infections.

Comparisons of mRNA levels of each candidate gene, between HAI and no HAI patients, at day 1 and at day 2–4, are presented on Fig 2.

#### Pro-inflammatory genes: IL1B and TNF (Fig 2A and 2B)

In our cohort of patients admitted to PICU, the whole blood mRNA levels of *TNF* were not different between those who did or did not developed HAI. Despite similar levels on admission, we observed a significantly higher expression for *IL1B* at day 2–4 in patients who developed HAI.

#### Compensatory anti-inflammatory response: IL10 and CD3D (Fig 2C and 2D)

At day 1 on PICU admission, *IL10* mRNA expression level was higher in patients that subsequently developed HAI (*p* = 0.03). This difference of expression remained statistically significant at day 2–4 (*p* = 0.03). Patients who developed HAI also exhibited a decreased expression of *CD3D* at both time points. This difference was statistically significant at day 1 (*p* = 0.04).

#### Apoptosis-related genes: BID and BCL2 (Fig 2E and 2F)

On PICU admission, the anti-apoptotic *BCL2* gene expression was significantly lower in patients who developed HAI than in those who did not. This difference was no longer present at day 2–4. In contrast, the pro-apoptotic *BID* gene expression increased over time (S1 Fig) and was significantly more expressed in patients who develop HAI at day 2–4.

#### Area under the receiver operating characteristic curve for predicting HAI

Each gene was tested using Receiver Operating Characteristic statistics to evaluate its performance in predicting HAI (Table 3). At day 1, *IL10*, *CD3D* and *BCL2* had similar performances, similar to the one obtained for WBC (AUC 0.71). The best performances were obtained for *BID*, *IL10* and *IL1B* at day 2–4, with AUC between 0.78 and 0.81 (*p*<0.05). As patients who contracted an HAI before day 2–4 were excluded, these results suggest that a panel of these biomarkers might provide useful discrimination in predicting which patients might develop HAI.

**Table 3:** Areas under curve for predicting hospital-acquired infection occurrence.

	Day 1		Day 2–4	
	AUC	95% CI	AUC	95% CI
***IL1B***	0.54	0.33–0.75	0.81*	0.55–1.06
***TNF***	0.56	0.35–0.76	0.60	0.32–0.88
***IL10***	0.72*	0.52–0.93	0.80*	0.59–1.00
***CD3D***	0.70*	0.51–0.89	0.67	0.41–0.93
***BCL2***	0.72*	0.54–0.89	0.58	0.31–0.85
***BID***	0.56	0.38–0.74	0.78*	0.51–1.04
**WBC**	0.71*	0.51–0.91	0.63	0.37–0.89

AUC: area under curve; CI: 95% confidence interval.

*: *p*<0.05.

#### Correlation study between biomarkers expression levels and disease severity indicators

Correlation analysis was performed between gene expression levels and parameters known to be associated with disease severity: PELOD score, ICU length of stay and hospital length of stay. In our cohort, we observed no significant correlation between our candidate biomarker expression and severity indicators **(S3 Table).**

Children who developed HAI were more likely to have genetic or chromosomal abnormalities (7/13 (54%)) than those who did not develop HAI (4/30 (13%), *p* = 0.01), suggesting a potential confounding factor **(Table 1)**. As shown on **S2 Fig**, we observed significant differences of gene expression levels between patients with and without chromosomal abnormalities: at day 1 for *IL10*, *CD3D* and *BID*, and at day 2–4 for *IL1B*, *TNF* and *BID*.

## Discussion

HAI have been associated to a higher morbidity of ICU patients both in adults [18,19] and children [20]. Interventions to prevent HAI could have a major impact in both personal (long term complications) and community (health costs) outcomes. However, such interventions would require tools to identify at-risk patients, which are currently lacking. As the host response plays a critical role in the risk of injury-induced immunosuppression, we assessed candidate host response biomarkers to determine their association with the occurrence of HAI: *IL1B*, *TNF*, *CD3D*, *IL10*, *BID* and *BCL2*.

*IL1B* and *TNF* are prototypical pro-inflammatory cytokines that orchestrate the inflammatory response. These two cytokines have been associated with mortality and HAI in several inflammatory situations [21,22]. In our study, we observed interesting performances for *IL1B*, especially when assessed at day 2–4 after admission. *TNF* and *IL1B* have been associated to prognosis and severity in several studies in adults. In our cohort, there was no significant correlation between relative gene expression and markers of disease severity.

One of the most promising markers in our study was *IL10*, with significant increase of expression in HAI patients as soon as at day 1. IL-10 is a prototypical anti-inflammatory cytokine which inhibits the production of IFNγ, decreases antigen presentation and promotes a Th2 pattern. Its main targets are lymphocytes and antigen presenting cells [23]. Elevated levels of IL-10 protein have been found in children after sepsis [24], trauma [24,25] or major surgery [26]. These high levels of IL-10 protein have been associated to the occurrence of HAI [27,28]. Here, we showed for the first time that the quantification of *IL10* mRNA expression in whole blood samples is also associated to HAI occurrence after PICU admission.

In a matched case-control study, Hinrichs *et al*. identified that a combination of three biomarkers–*CD3D*, *IL1B* and *TNF*–was the best predictor of post-operative sepsis in an adult cohort of patients [29], with a specificity of 90% and a sensitivity of 85%. *CD3D* is the gene coding the delta subunit of the CD3 molecule, which plays a crucial role in T lymphocyte signal transduction after TCR engagement. Mutation in *CD3D* gene are responsible for some rare cases of severe combined immune deficiency syndrome [30], characterized by a failure of T cell differentiation in thymus and an adaptive immune dysfunction. *CD3D* was also recently identified in a microarray study comparing the expression profile of whole blood samples from surgical patients diagnosed with either SIRS or sepsis. *CD3D* expression was again lower in septic patients [31]. Here, we confirm in children that *CD3D* might be a good biomarker candidate to predict HAI occurrence in ICU patients admitted to PICU with SIRS.

Despite higher total white cell counts, patients that developed HAI tend to have a lower lymphocyte counts. Therefore, the lower expression of *CD3D* in whole blood may reflect the lymphopenia that occurs after sepsis [32] or SIRS [33] and emphasize the role of T-cell response in HAI occurrence.

Apoptosis of immune cells, in particular lymphocytes, is recognized as a core feature of sepsis pathophysiology. We and other had previously shown that apoptosis related genes were modulated in adult septic patients [5,11]. We hypothesized that *BID* and *BCL2*, which are respectively key pro- and anti-apoptotic genes would therefore be good candidate biomarkers to predict the risk of HAI. Our results suggest that paediatric critically ill patients exhibit in the days following PICU admission a pro-apoptotic profile, similar to the pattern seen in adult septic patients [5]. As we measured gene expression from whole blood samples, we cannot speculate on the immune cell subset impacted by this phenomenon. However, several reports highlighted the role of sepsis in lymphocyte apoptosis [32]. Lymphopenia has been found in children with septic shock [34], similarly to adults. In children with multiple organ failure, a prolonged lymphopenia (<1G/L for > 7 days) was associated with HAI [35]. Our results suggest that the quantification of mRNA apoptosis-related biomarkers may detect this enhanced lymphocyte apoptosis and identify patients at higher risk of HAI.

In this study, we have shown that in children over 28 days of age, *IL1B*, *IL10*, *CD3D*, *BCL2* and *BID* are differentially expressed in children who develop HAI. These genes could be used as a biomarker panel to stratify children at risk of HAI. Increasing evidence has linked up-regulation of apoptotic and anti-inflammatory markers with poor outcome from sepsis and secondary infection [1,10–12,26,36–39]. This main mechanism may be linked to lymphocyte apoptosis and anergy. A recent study reported decreased T-cell *ex vivo* PHA-induced production of IFNγ, IL-2 and IL-10 in children with septic shock that went on to develop persistent or nosocomial infection compared with septic shock children who did not [40]. Our findings provides further evidence that immunosuppression may occurs in PICU and be associated with secondary infections in children.

Recombinant IL-7 and PD-1 blockade are potential new immunomodulatory therapies, for which risk stratification biomarker panels could help predict which patients might derive benefit. If our findings are confirmed in larger groups of patients, then such biomarker guided- strategies could be used as a novel approach to prevent HAI in critically ill children on intensive care. The availability of rapid automated molecular diagnostic tools now offer a real possibility of developing these assays as a bedside test in critically ill children.

This pilot study has some limitations. First, this study has a small sample size population and therefore we were unable to assess the predictive power of our biomarkers. The population was heterogeneous in terms of patient age and the presenting problems. Future analysis should group patients into separate categories such as surgical, sepsis and medical, with a larger number of patients for a sufficiently powered study. In our study, chromosomal or genetic abnormalities may be a confounding factor for HAI occurrence. This aspect has to be specifically taken into account in future validation studies in larger cohorts.

## Conclusions

Despite meticulous infection control policies, HAI is a common complication of intensive care, and is favored by critical illness induced immunosuppression. The development of HAI increases length of hospital stay, therefore prevention would lead to significant patient benefit and reduction in health care costs. The early identification of children at risk of HAI can help provide risk stratification parameters for pre-emptive immunomodulatory therapies which restore immune function and prevent the development of HAI. In this small pilot study of critically ill children we demonstrate for the first time, using qPCR, that a panel of immune markers might provide such a novel stratification tool. These findings require confirmation in a larger cohort, to determine if this panel may be useful for patient stratification in future clinical trials of immunomodulatory drugs.

## Supporting Information

S1 Fig Comparison of gene expression levels in individual patients between day 1 and day 2–4 for HAI and no HAI patients. No HAI patients (Clear): n = 14 (except IL10: n = 13); HAI patients (Grey): n = 6. Gene expression levels of (A) *IL1B*, (B) *TNF*, (C) *IL10*, (D) *CD3D*, (E) *BCL2* and (F) *BID* are expressed as Calibrated Normalized Relative Quantity using *PPIB* and *HPRT1* as reference genes. Expression levels on day 1 and day 2–4 were compared using paired Wilcoxon test and *p* <0.05 are indicated on plots. HAI: hospital-acquired infections
(TIF)

S2 Fig Comparison of gene expression levels between patients with and without chromosomal abnormalities. No chromosomal abnormality patients (Clear): n = 31 on day 1 and n = 14 on day 2–4 (except for IL10, n = 30 on day 1); chromosomal abnormality patients (Grey): n = 11 on day 1 and n = 7 on day 2–4. Gene expression levels of (A) *IL1B*, (B) *TNF*, (C) *IL10*, (D) *CD3D*, (E) *BCL2* and (F) *BID* are expressed as Calibrated Normalized Relative Quantity using *PPIB* and *HPRT1* as reference genes. Expression levels between patients with and without chromosomal abnormalities were compared using Mann-Whitney test and *p* <0.05 are indicated on plots.
(TIF)

S1 Table Primer and probe sequences. Designs of primers and probes used for the messenger RNA quantification by RT-qPCR of genes of interest and reference genes.
(PDF)

S2 Table Characteristics of the 21 paediatric patients considered for analysis on Day 2–4 according to hospital-acquired infection occurrence (PDF)

S3 Table Spearman correlation coefficients between mRNA expression levels of candidate biomarkers and PELOD, ICU and hospital length of stay. (PDF)
