# Supplementary figures and images for: Evaluation of mRNA Biomarkers to Identify Risk of Hospital Acquired Infections in Children Admitted to Paediatric Intensive Care Unit
Source: PLoS One. 2016 Mar 25;11(3):e0152388. doi: 10.1371/journal.pone.0152388 (PMC4807819; doi:10.1371/journal.pone.0152388)

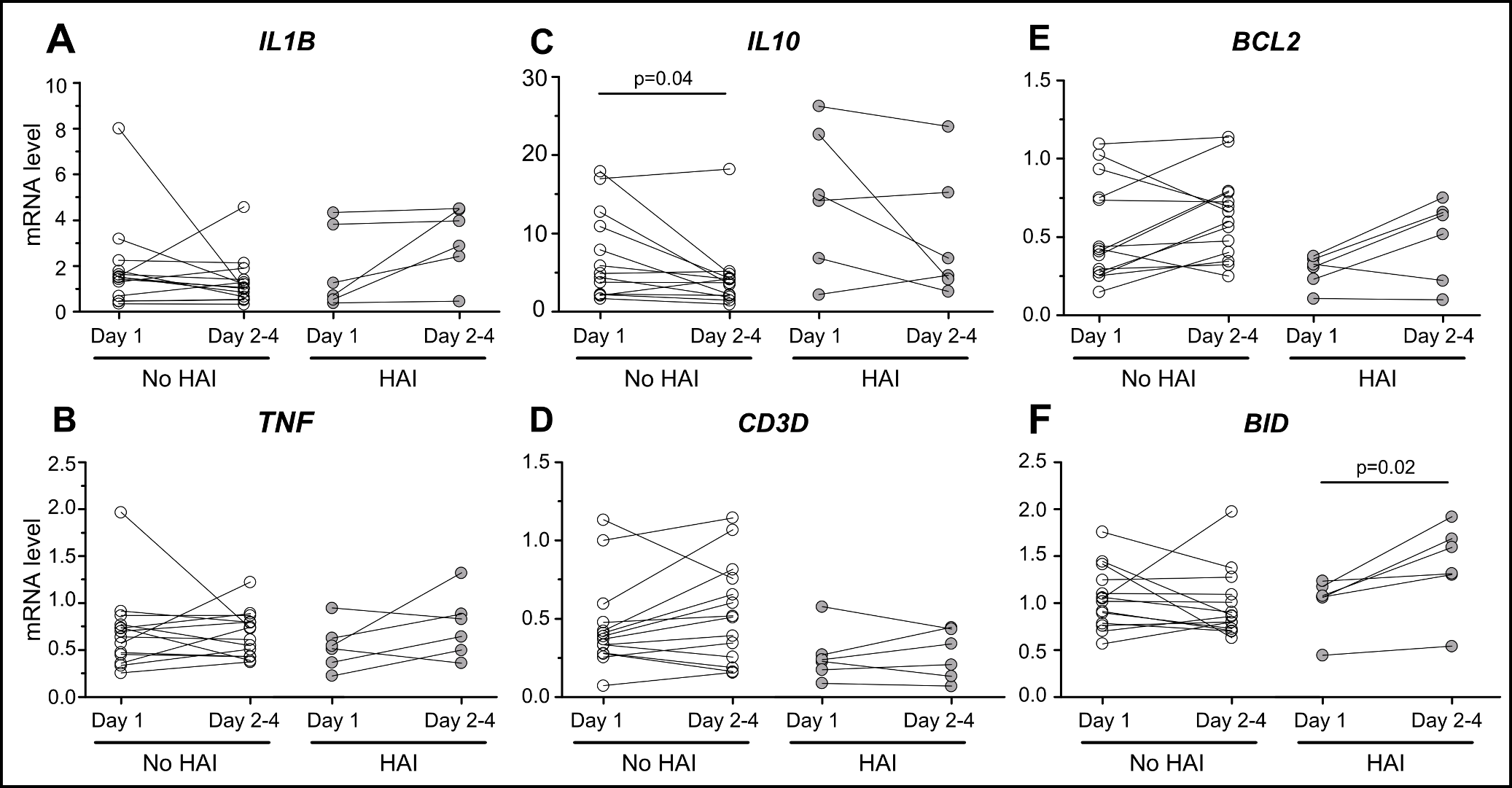

Supplement: S1 Fig — No HAI patients (Clear): n = 14 (except IL10: n = 13); HAI patients (Grey): n = 6. Gene expression levels of (A) IL1B, (B) TNF, (C) IL10, (D) CD3D, (E) BCL2 and (F) BID are expressed as Calibrated Normalized Relative Quantity using PPIB and HPRT1 as reference genes. Expression levels on day 1 and day 2–4 were compared using paired Wilcoxon test and p <0.05 are indicated on plots. HAI: hospital-acquired infections (TIF) [file pone.0152388.s001.tif]

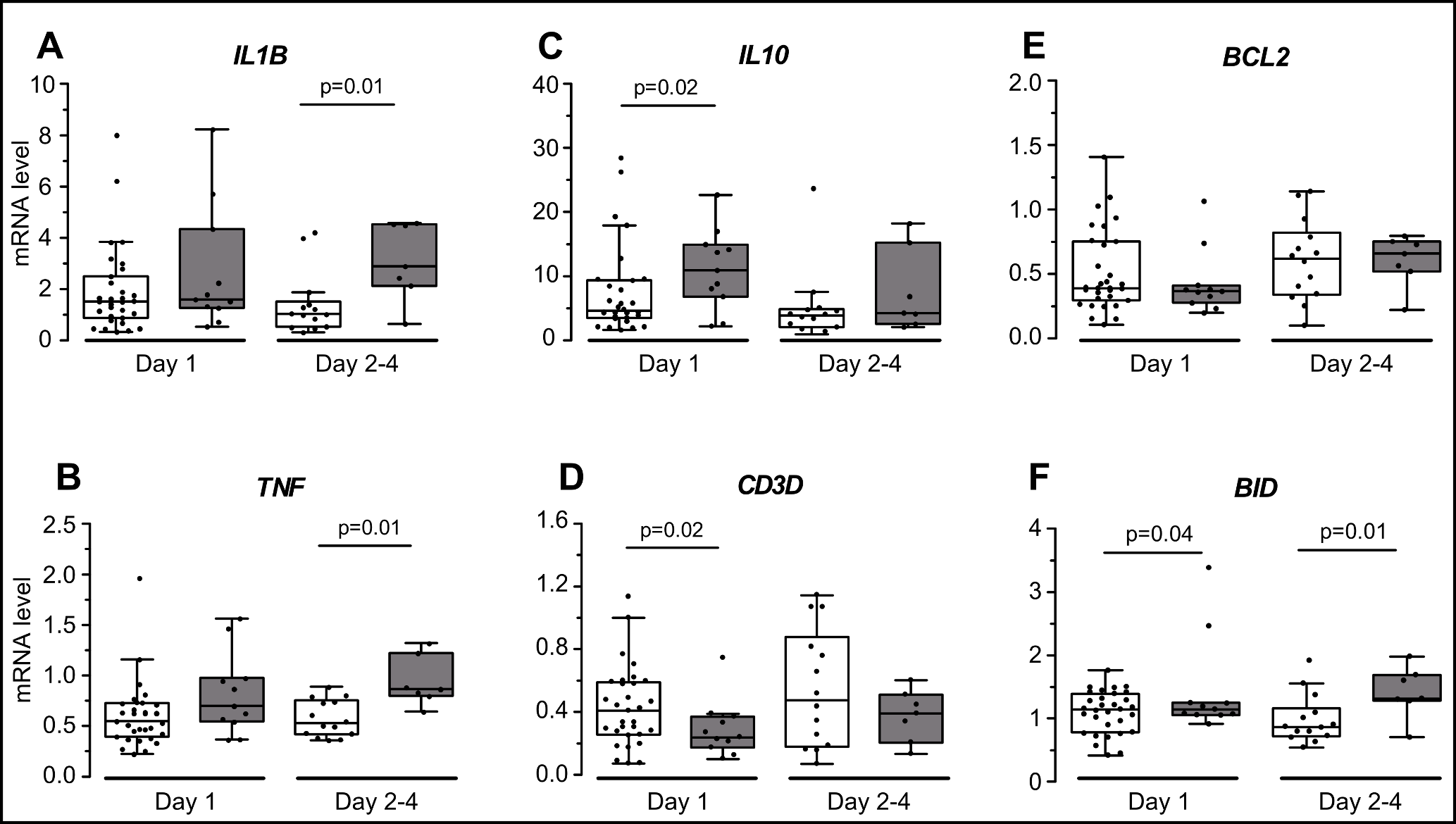

Supplement: S2 Fig — No chromosomal abnormality patients (Clear): n = 31 on day 1 and n = 14 on day 2–4 (except for IL10, n = 30 on day 1); chromosomal abnormality patients (Grey): n = 11 on day 1 and n = 7 on day 2–4. Gene expression levels of (A) IL1B, (B) TNF, (C) IL10, (D) CD3D, (E) BCL2 and (F) BID are expressed as Calibrated Normalized Relative Quantity using PPIB and HPRT1 as reference genes. Expression levels between patients with and without chromosomal abnormalities were compared using Mann-Whitney test and p <0.05 are indicated on plots. (TIF) [file pone.0152388.s002.tif]
