# Supplementary material for: Evaluation of mRNA Biomarkers to Identify Risk of Hospital Acquired Infections in Children Admitted to Paediatric Intensive Care Unit
Source: PLoS One. 2016 Mar 25;11(3):e0152388. doi: 10.1371/journal.pone.0152388 (PMC4807819; doi:10.1371/journal.pone.0152388)
Supplement: S1 Table — Designs of primers and probes used for the messenger RNA quantification by RT-qPCR of genes of interest and reference genes. (PDF) [file pone.0152388.s003.pdf]

Evaluation of mRNA biomarkers to identify risk of hospital acquired infections in children admitted to paediatric intensive care unit

Estelle Peronnet, Kha Nguyen, Elisabeth Cerrato, Rathi Guhadasan, Fabienne Venet, Julien Textoris, Alexandre Pachot, Guillaume Monneret and Enitan Delphine Carrol

S1 Table. Primer and probe sequences. Designs of primers and probes used for the messenger RNA quantification by RT-qPCR of genes of interest and reference genes.

Gene name	Accession number	Sequences
CD3D	NM_000732<br>NM_001040651	5'-GCTCTGTTGAGGAATGACC-3' (sens)<br>5'-GAAGGGAAGGTACAGTTGG-3' (antisens)<br>5'-GGCTCGGAACAAGTGAACCTG-3' (probe)
HPRT1	NM_000194	5'-CCAAAGATGGTCAAGGTCGC-3' (sens)<br>5'-GACACAAACATGATTCAAATCC-3' (antisens)<br>5'-CAAGTTTGTTGTAGGATATGCCC-3' (probe)
IL10	NM_000572	5'-AGAACCAAGACCCAGACATC-3' (sens)<br>5'-CATTCTTCACCTGCTCCAC-3' (antisens)<br>5'-TCTTCCCTGTGAAAACAAGAGC-3' (probe)
IL1B	NM_000576	5'-GATGATAAGCCCACTCTACAG-3' (sens)<br>5'-CTGCTTGAGAGGTGCTGATG-3' (antisens)<br>5'-CTGGAATTTGAGTCTGCCCAG-3' (probe)
PPIB	NM_000942	5'-GGAGATGGCACAGGAGGAAAGA-3' (sens)<br>5'-GGGAGCCGTTGGTGTCTTTG-3' (antisens)<br>5'-GGTGAGCATGGCCAACGCAGG-3' (probe)
TNF	NM_000594	5'-CCGAGTGACAAGCCTGTAG-3' (sens)<br>5'-CCTCTGATGGCACCACCAG-3' (antisens)<br>5'-GGCAGCTCCAGTGGCTGAAC-3' (probe)

CD3D: CD3d molecule, delta (CD3-TCR complex),
HPRT1: hypoxanthine phosphoribosyltransferase 1,
IL10: interleukin 10,
*IL1*B: interleukin 1, beta,
PPIB: peptidylprolyl isomerase B (cyclophilin B),
TNF: Tumor necrosis factor.
