# Supplementary material for: Evaluation of mRNA Biomarkers to Identify Risk of Hospital Acquired Infections in Children Admitted to Paediatric Intensive Care Unit
Source: PLoS One. 2016 Mar 25;11(3):e0152388. doi: 10.1371/journal.pone.0152388 (PMC4807819; doi:10.1371/journal.pone.0152388)
Supplement: S2 Table — (PDF) [file pone.0152388.s004.pdf]

Evaluation of mRNA biomarkers to identify risk of hospital acquired infections in children admitted to paediatric intensive care unit

Estelle Peronnet, Kha Nguyen, Elisabeth Cerrato, Rathi Guhadasan, Fabienne Venet, Julien Textoris, Alexandre Pachot, Guillaume Monneret and Enitan Delphine Carrol

S2 Table. Characteristics of the 21 paediatric patients considered for analysis on Day 2-4 according to hospital-acquired infection occurrence.

Variable	HAI (N=7)	No HAI (N=14)	Total (N=21)	*p* value
**Demographics**				
Gender – Male, n (%)	4 (57)	8 (57)	12 (57)	1.00
Age (Months), median [IQR]	12 [8.5 - 29]	9.5 [4 - 29]	11 [5 - 34]	0.63
**Admission data**				
Reason for admission				1.00
Cardiac surgery, n (%)	4 (57)	7 (50)	11 (52)	
Other surgery, n (%)	0 (0)	1 (7)	1 (5)	
Sepsis, n (%)	2 (29)	4 (29)	6 (29)	
Other, n (%)	1 (14)	2 (14)	3 (14)	
Comorbidities				
Congenital heart disease, n (%)	0 (0)	5 (36)	5 (24)	0.12
Non cardiac congenital disease, n (%)	1 (14)	1 (7)	2 (10)	1.00
Chromosomal abnormality, n (%)	4 (57)	3 (21)	7 (33)	0.16
PELOD, median [IQR]	12 [11 - 20]	11 [11 - 15][a]	11 [11 - 20][b]	0.27
Cardiopulmonary bypass (CPB), n (%)	4 (57)	7 (50)	11 (52)	1.00
Dexamethasone (Pre, post, during CPB), n (%)	3 (75)[c]	5 (71)[d]	8 (73)[e]	1.00
PIM2 score, median [IQR]	0.042 [0.015 - 0.066][c]	0.056 [0.036 - 0.081][d]	0.054 [0.028 - 0.079][e]	0.35
**Treatments**				
ATB prior to admission, n(%)	4 (57)	7 (58)[a]	11 (58)[b]	1.00
ATB during admission, n(%)	7 (100)	14 (100)	21 (100)	1.00
Blood transfusion, n(%)	4 (57)	7 (54)[f]	11 (55)[g]	1.00
**Biological data day 1**				
WCC (10^9/L), median [IQR]	17 [14 - 25]	10 [8.2 - 12]	11 [8.6 - 17]	**<u>0.04</u>**
Lymphocytes (10^9/L), median [IQR]	1.0 [0.8 - 1.2]	1.6 [1.1 - 2.6]	1.3 [1.0 - 2.6]	0.23
Lactate (mmol/L), median [IQR]	1.4 [0.9 - 1.9][h]	1.1 [0.8 - 1.7][f]	1.1 [0.8 – 1.9][b]	0.20
C reactive protein (mg/L)	25 [7.9 - 60][h]	6.1 [4 - 28]	8.4 [4.0 - 41]	0.64
**Biological data day 2-4**				
WCC (10^9/L), median [IQR]	11 [9.8 - 20]	11 [7.7 - 14]	11 [8.9 - 15]	0.11
Lymphocytes (10^9/L), median [IQR]	2.9 [2.2 - 3.3]	2.3 [1.6 – 4.0]	2.4 [1.7 - 3.6]	0.79
C reactive protein (mg/L), median [IQR]	25 [18 - 59]	23 [9.2 - 76]	23 [12 - 64]	0.74
**Risk factors**				
Invasive devices at admission				
Intubation, n (%)	7 (100)	14 (100)	21 (100)	1.00
Central venous line, n (%)	6 (86)	10 (71)	16 (76)	0.62
Urinary catheter, n (%)	6 (86)	11 (79)	17 (81)	1.00
**Outcomes**				
Mortality, n (%)	0 (0)	2 (14)	2 (9.5)	0.53
ICU length of stay (Days), median [IQR]	14 [5 - 18]	6 [4 - 8]	6 [4 - 14]	0.11
Hosp. length of stay (Days), median [IQR]	31 [28 - 43]	12 [8 - 19]	16 [9 - 29]	**<u><0.001</u>**
Ventilation duration (Days), median [IQR]	11 [5 - 14]	5 [3 - 7]	6 [3 - 8]	0.08

[a] n=12, [b] n=19, [c] n=4, [d] n=7, [e] n=11, [f] n=13, [g] n=20, [h] n=6

HAI and no HAI groups were compared using Mann-Whitney test for continuous variables and Fisher’s exact test for categorical variables. *p* values <0.05 are bold.

HAI: Hospital-Acquired Infection. Hosp: hospital. PELOD: Pediatric Logistic Organ Dysfunction. CPB: Cardiopulmonary bypass. PIM2: Pediatric Index for Mortality. ATB: antibiotics.. WCC: White cells count. ICU: Intensive Care Unit.
