# Supplementary material for: Evaluation of mRNA Biomarkers to Identify Risk of Hospital Acquired Infections in Children Admitted to Paediatric Intensive Care Unit
Source: PLoS One. 2016 Mar 25;11(3):e0152388. doi: 10.1371/journal.pone.0152388 (PMC4807819; doi:10.1371/journal.pone.0152388)
Supplement: S3 Table — (PDF) [file pone.0152388.s005.pdf]

**Evaluation of mRNA biomarkers to identify risk of hospital acquired infections in children admitted to paediatric intensive care unit**

Estelle Peronnet, Kha Nguyen, Elisabeth Cerrato, Rathi Guhadasan, Fabienne Venet, Julien Textoris, Alexandre Pachot, Guillaume Monneret and Enitan Delphine Carrol

**S3 Table. Spearman correlation coefficients between mRNA expression levels of candidate biomarkers and PELOD, ICU and hospital length of stay.** Correlations with r>0.8 were considered significant.

| **Gene name** | **Day 1** | | | **Day 2-4** | | |
|---|---|---|---|---|---|---|
| | **PELOD** | **ICU length of stay** | **Hospital length of stay** | **PELOD** | **ICU length of stay** | **Hospital length of stay** |
| ***IL1B*** | -0.124 | -0.333 | -0.052 | 0.399 | 0.276 | 0.496 |
| ***TNF*** | -0.185 | -0.133 | -0.098 | 0.056 | 0.237 | 0.321 |
| ***IL10*** | 0.531 | 0.002 | 0.191 | 0.632 | 0.333 | 0.295 |
| ***CD3D*** | -0.395 | -0.038 | -0.269 | -0.433 | -0.124 | 0.045 |
| ***BCL2*** | -0.316 | -0.101 | -0.269 | -0.420 | -0.152 | 0.135 |
| ***BID*** | -0.244 | -0.330 | -0.164 | 0.500 | 0.438 | 0.502 |
